# King Edward's Fund and the Evelina Hospital

**Published:** 1912-02-17

**Authors:** Savile Crossley, John G. Griffiths

**Affiliations:** Honorary Secretaries. 7 Walbrook, London, E.C.


*To the Editor of* The Hospital.

### KING EDWARD'S FUND AND THE EVELINA HOSPITAL.

*To the Editor of* The Hospital.

Sir,—We venture to trouble you with the following observations in order to remove a misapprehension as to the attitude of King Edward's Hospital Fund which may arise from the published letter of the Chairman of the Evelina Hospital. In his statement of the reasons for the refusal of a grant by the King's Fund the Chairman presents the necessary result of a deliberate act of his committee as if it were the arbitrary act of the Fund; he has omitted to mention that in the Fund's official notification of the reasons why no grant could be made it was stated that "no application could be considered until the regulations of the revised uniform system of hospital accounts were complied with by the replacement of the sum of £3,620 transferred from the General Fund to the Samaritan Fund."

The revised uniform system of hospital accounts was adopted in 1906 by the King's Fund, the Hospital Sunday Fund, and the Hospital Saturday Fund, with the concurrence of a representative committee of hospital secretaries, in order to enable the yearly financial operations of the various London hospitals to be correctly compared. In fairness both to the hospitals generally and to the subscribing public, the King's Fund is bound to see that the system is observed by institutions applying for grants.

The regulation which the Evelina Committee have ignored is one not of form but of substance, and the effect on the accounts is important. A sum of £3,620 which they have full power to apply to the general purposes of the hospital, particularly to meet a deficit in working, is shown on their balance-sheet under the heading specially reserved for funds which cannot be applied to meet a deficit in working or other general purposes. Similarly the income from this considerable sum is excluded from the income and expenditure account.

If, therefore, these accounts are published as being in accordance with the revised uniform system, the balance sheet is to this extent incorrect, and the income and expenditure account is incomplete; if these accounts are not intended to be interpreted according to the uniform system, then the Hospital Committee are confessedly not observing the system, and are making the hospital ineligible for a grant. There is thus nothing arbitrary in the King's Fund action; it is the automatic result of the free choice by the Hospital Committee of a course which inevitably produces this dilemma.

The Fund appreciates the courteous terms in which the Chairman of the Hospital refers to its previous relations with the Fund; but we must point out that the Fund has not interfered with the management of the hospital. The customary stipulation that there should be an inquiry officer can hardly be so described, and if it could, it is not the point at issue. Even with regard to the method of publishing the accounts, the Fund is only concerned because the receipt of a grant would imply that the accounts were in the recognised form which enables the financial position and operations of one hospital to be compared with those of other hospitals. Unfortunately the accounts of the Evelina Hospital are not in this form.—We are, Sir, your obedient servants,

(Signed) SAVILE CROSSLEY,
JOHN G. GRIFFITHS,
Honorary Secretaries.

7 Walbrook, London, E.C.
February 8.

